# Ventriculoperitoneal Shunt Failure and Cerebrospinal Fluid Protein: A Meta-Analysis and Systematic Review

**DOI:** 10.7759/cureus.54362

**Published:** 2024-02-17

**Authors:** Kyle M Rei, Muhammad S Ghauri, Mohammed B Uddin, Javed Siddiqi

**Affiliations:** 1 Neurosurgery, California University of Science and Medicine, Colton, USA; 2 Neurosurgery, Arrowhead Regional Medical Center, Colton, USA; 3 Neurosurgery, Desert Regional Medical Center, Palm Springs, USA; 4 Neurosurgery, Riverside University Health System Medical Center, Moreno Valley, USA

**Keywords:** ventriculoperitoneal shunt complications/malfunction, vp shunt complication, csf protein, hydrocephalus, ventriculoperitoneal (vp) shunt

## Abstract

Ventriculoperitoneal shunts (VPS) are used to manage hydrocephalus but suffer from high failure rates. Our objectives were to (1) conduct a meta-analysis to objectively weigh this conflicting evidence, and (2) conduct a systematic review compiling and synthesizing what is known about the association between CSF proteins and shunt failure. A literature search was performed in compliance with Preferred Reporting Items for Systematic Reviews and Meta-Analyses guidelines. The Embase, PubMed, and CENTRAL databases were searched from inception to June 2023. The articles were screened based on the inclusion criteria. A meta-analysis was conducted using R statistical software (R Foundation for Statistical Computing, Vienna, Austria); heterogeneity, subgroup, sensitivity, risk of bias, and publication bias analyses were performed.

Thirty-one studies were selected for the systematic review, of which eight were selected for the meta-analysis. Perioperative CSF protein level was compared between 351 shunt failures and 1,094 shunt survivals; the mean difference of 24.37 mg/dL favoring shunt failure was significant (95% confidence interval=2.44-46.29 mg/dL). Our systematic review yielded a hypothesized pathogenesis: proteins attached to imperfections in the shunt surface lead to secondary attachment of cells, particularly astrocytes, and tertiary attachment of ependymal cells and the choroid plexus. Owing to the limitations of this meta-analysis, including lack of robustness due to missing data, heterogeneity, and certainty of the evidence, future research is needed to better understand the relationship between perioperative CSF protein levels and shunt failure.

## Introduction and background

The ventriculoperitoneal shunt (VPS) is a vital tool in the management of hydrocephalus (HCP), which is the abnormal accumulation of cerebrospinal fluid (CSF) in the brain [1]. These shunts function by diverting CSF from the ventricular system of the brain to the peritoneal cavity; over 30,000 units are implanted annually in the US. Unfortunately, 11-25% of shunts fail within the first year of placement and often require multiple shunt revisions throughout a patient's lifetime [2]. Shunt failure, which is defined herein as encompassing all types of malfunction, most commonly includes obstruction (proximal, valvular, or distal) and infection [3].

The role of CSF proteins in shunt failure has been a topic of contention in literature. Although it has long been assumed that elevated CSF protein levels increase the risk of shunt failure, this assertion lacks robust evidence and has been scrutinized as developing from hearsay [4]. This conventional wisdom is based on the fact that CSF proteins can adhere to shunt surfaces [5], serve as a nexus for secondary cellular attachment [6], and increase CSF viscosity [7]. While several studies have found an association between CSF protein levels and shunt failure, [8-10] others have found no association [3,11-16]. Our objectives were to (1) conduct a meta-analysis to objectively weigh this conflicting evidence, and (2) conduct a systematic review compiling and synthesizing what is known about the association between CSF proteins and shunt failure.

## Review

Methods

Ethical Guidance

The reporting of this meta-analysis and systematic review was guided by the Preferred Reporting Items for Systematic Reviews and Meta-Analyses (PRISMA) Statement.

Selection Criteria

The inclusion criteria for the meta-analysis were as follows: (1) date range: inception to June 30, 2023; (2) domain: neurosurgical management of elevated intracranial pressure via VPS; (3) study type: randomized controlled trial, prospective cohort, retrospective cohort, or case-control; (4) population: all demographics and HCP etiologies; (5) exposure: elevated CSF protein; (6) outcome: shunt failure; and (7) comparator: shunt survival. This systematic review included studies investigating the effects of CSF proteins on shunt function. The exclusion criteria were as follows: (1) language: non-English; (2) publication type: abstract; and (3) study type: case study, review, or animal study.

One synthesis was performed in the meta-analysis that compared mean perioperative CSF protein levels between the shunt failure and survival groups. In the systematic review, three syntheses were performed according to the research design: (1) benchtop perfusion models, (2) explant analyses, and (3) CSF analyses.

Search Strategy

The Embase, PubMed, and Cochrane Central Register of Controlled Trials (CENTRAL) databases were searched using the strategy outlined in Table 1. No filters or limits were applied in the search. The search strategy was calibrated by reviewing 50 relevant terms.

**Table 1:** Search strategy CENTRAL: Cochrane Central Register of Controlled Trials

Step	Search terms	Embase	PubMed	CENTRAL
1	vp OR “ventriculoperitoneal” OR csf OR “cerebrospinal fluid”	352,317	219,754	14,351
2	shunt	107,628	88,224	3,246
3	#1 AND #2	18,068	17,146	414
4	failure OR malfunction OR obstruction OR occlusion OR survival OR complication* OR flow	8,313,350	7,315,078	489,054
5	#3 AND #4	11,781	11,823	258
6	protein	6,790,692	8,276,079	120,117
7	#5 AND #6	823	678	13

Process

Records were exported from each database and compiled into Excel files. After removing duplicates, the records were independently screened by two reviewers. Disagreements were resolved through discussion between KR and MU. If a referee was required, then the MG was consulted. Two reviewers, KR and MG, extracted the data into an Excel file and a separate reviewer (MU) verified the accuracy of the extracted data. Automation tools were not used in the study.

Data Items

Meta-analysis data were collected for the perioperative CSF protein levels (mean and standard deviation (SD)) and the outcome of shunt failure for any reason versus shunt survival. Studies that did not provide data for this dichotomous outcome were excluded from the meta-analysis and discussed in a systematic review. Additional data were collected on country, research design, n, mean follow-up period, age, sex, HCP etiology, definition of shunt failure, and failure rate.

Risk of Bias Assessment

The risk of bias was assessed using the Risk Of Bias In Non-randomized Studies - of Exposures (ROBINS-E) tool. Two reviewers (KR and MG) collaboratively provided ratings for each study across seven domains: (1) confounding factors, (2) exposure measurement, (3) participant selection, (4) interventions, (5) missing data, (6) outcome measurement, and (7) reporting results. Disagreements were resolved through discussion between KR and MG. Automation tools were not used in the study.

Effect Measures

The perioperative CSF protein levels were reported as mean and SD; the mean difference (MD) was used in the synthesis of the results.

Synthesis Methods

Studies were considered eligible for inclusion in the meta-analysis if the following data were available: perioperative CSF protein levels in the shunt survival vs. failure groups. Studies lacking data for both outcome groups were considered ineligible for inclusion. Additionally, a follow-up of at least two months was required to provide an adequate opportunity for shunt failure to occur. For the systematic review, studies were considered eligible for inclusion in one of three syntheses based on the research design.

Studies [10] reporting multiple groups that required combination for inclusion in the meta-analysis were performed according to the Cochrane guidelines [17]. For studies with missing SD data [8,9,13], estimation methods were used to calculate the SD from the range [18], confidence interval, and p-value [17]. For comparison, studies [9,13,16] reporting CSF protein levels in g/L were converted to mg/dL.

Meta-analysis study-level characteristics are reported in table format: (1) author and year, (2) Grading of Recommendations Assessment, Development, and Evaluation (GRADE), (3) age, (4) sex, (5) HCP etiology, (6) failure definition, (7-9) shunt failure group n and CSF protein mean/SD, and (10-12) shunt survival group n and CSF protein mean/SD.

Systematic review study-level characteristics are reported in a table format: (1) author and year, (2) GRADE, (3) country, (4) study category, (5) research question, (6) methods, (7) population, (8) HCP etiology, (9) measures, and (10) findings.

Data Analysis

Statistical analysis was performed using R statistical software (R Foundation for Statistical Computing, Vienna, Austria) with the meta package. Continuous outcomes were assessed using the Metacont function. A random-effects model was used to report the MDs and the associated 95% confidence intervals. Heterogeneity was assessed using I2 following the guidelines of the Cochrane Handbook for Systematic Reviews of Interventions: 0-40% might not be important, 30-60% may be moderate, 50-90% may be substantial, and 75-100% is considerable [17].

The causes of heterogeneity were explored using subgroup analysis. Subgroups according to age and HCP etiology were prospectively selected. Studies with neonates to early pediatric patients were subgrouped; this population has different etiologies and complications compared to adults with VPS [13,14]. Studies were subgrouped by HCP etiology; while the authors aimed to study the effect of CSF proteins on shunt failure, the true cause is unlikely to be univariate, and CSF composition can vary widely depending on HCP etiology.

A sensitivity analysis was conducted to assess the robustness of the synthesized results, despite the estimations used for SD due to missing data. To test this, we performed an additional analysis, excluding studies with missing data. This analysis was not conducted prospectively.

Publication bias was assessed using funnel plots and Egger regression tests with the metabias R function. To assess the certainty of the evidence, methods recommended by the GRADE system were used [19].

Results

Study Selection

**Figure 1:**
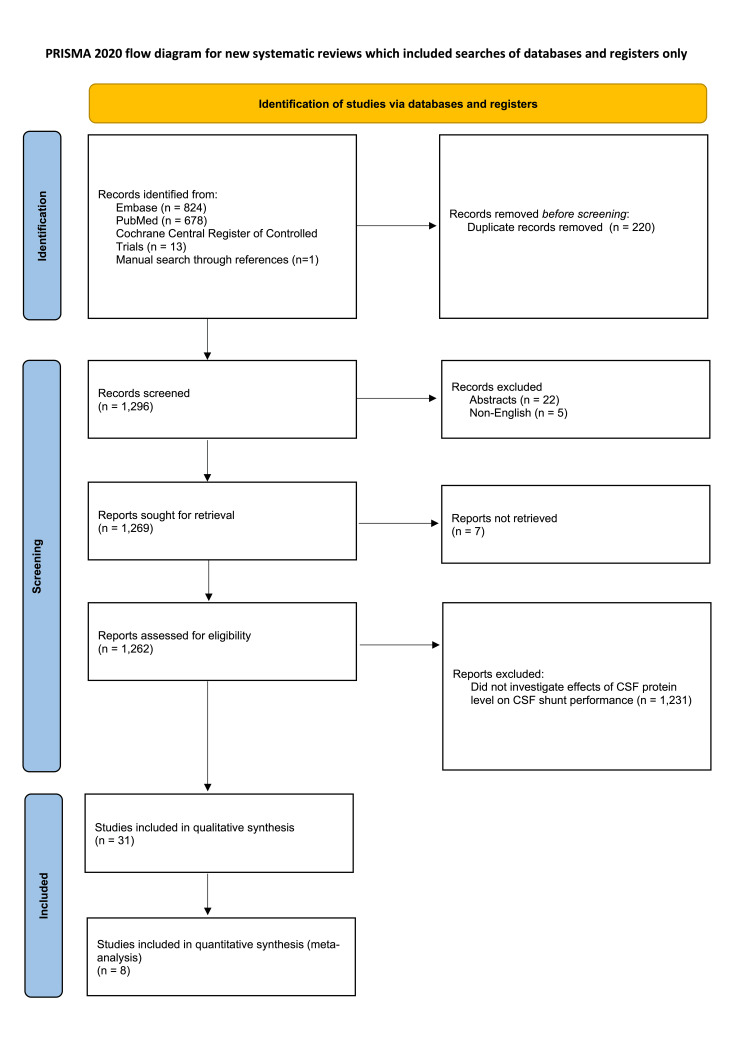
PRISMA flow diagram PRISMA: Preferred Reporting Items for Systematic Reviews and Meta-Analyses

The literature search yielded 1,515 records: Embase=823, PubMed=678, and CENTRAL=13 (Figure 1). An additional study was conducted through a manual search of references. After removing 220 duplicate records, 1,296 titles and abstracts were screened. After applying the inclusion and exclusion criteria, 31 studies were selected for qualitative synthesis [1-35]. These studies broadly fell into three categories: benchtop perfusion models (6 studies), explant analyses (6 studies), and CSF analyses (19 studies). Of these 31 studies, 8 met the inclusion criteria for the meta-analysis and were selected for quantitative analysis.

Study Characteristics

The study characteristics and results of the meta-analysis and systematic review are summarized in Tables 2, 3, respectively.

**Table 2:** Meta-analysis study characteristics GRADE: Grading of Recommendations Assessment, Development, and Evaluation; HCP: Hydrocephalus; TB: Tuberculous; CSF: Cerebrospinal fluid; SD: Standard deviation

						Shunt failure group			Shunt survival group		
Author, year	GRADE	Age mean (range)	Female	HCP etiology	Failure definition	n	Mean CSF protein (mg/dl)	SD CSF protein (mg/dl)	n	Mean CSF protein (mg/dl)	SD CSF protein (mg/dl)
Brydon et al., 1996 [13]	Low	3.9 (1d-14.9y)	45.26%	Mixed	Obstruction only	12	149	303	91	76	141
Ambekar et al., 2011 [8]	Low	15 (1-40)	39.62%	TB meningitis	All shunt failures (excluding infection and malposition)	70	311.85	731	137	134.84	180.17
Fulkerson et al., 2011 [14]	Low	26.5w (25-35w)	NA	Post-hemorrhagic	All shunt failures	19	237.07	141.99	39	197.9	165.4
Bir et al., 2017 [3]	Low	64 (25-86)	68.75%	Meningioma	All shunt failures	13	115.07	74.77	35	90.37	33.77
Kamat et al., 2018 [9]	Low	27.2 (NA)	52.54%	TB meningitis	Obstruction only	170	294	407	420	176	4.07
Richetta et al., 2019 [10]	Low	53.7 (NA)	55.00%	Brain tumor	All shunt failures	23	59.17	76.84	66	39.3	54.8
Kaestner et al., 2021 [15]	Low	50.4 (NA)	47.81%	Mixed	Obstruction only	32	73	64	242	68	108
Dong et al., 2022 [16]	Low	43.9 (11-77)	28.57%	Cryptococcal meningitis (HIV-negative)	All shunt failures (and 3 cases of post-operative epilepsy)	12	17.5	13.75	64	12	34.8

**Table 3:** Systematic review study characteristics A1: Inflammatory astrocyte phenotype; A2: Anti-inflammatory astrocyte phenotype; BSA: Bovine serum albumin; C3: Gene associated with label for A1 reactive astrocytes; ECP: Eosinophil cationic protein; EDN: Eosinophil-derived neurotoxin; EDX: Energy-dispersive X-ray; ELISA: Enzyme-linked immunosorbent assay; EMP1: Gene associated with label for A2 reactive astrocytes; ETO: Ethylene oxide; GRADE: Grading of Recommendations Assessment, Development and Evaluation; HCP: Hydrocephalus; IVH: Intraventricular hemorrhage; MMP: Matrix metalloproteinase; NPV: Nonprogrammable valve; PHH: Post-hemorrhagic hydrocephalus; PHVD: Posthemorrhagic ventricular dilation; PV: Programmable valve; qPCR: Quantitative polymerase chain reaction; SAH: Subarachnoid hemorrhage; SEM: Scanning electron microscopy; TB: Tuberculous; TEM: Transmission electron microscopy; VPS: Ventriculoperitoneal shunt

Author, year	GRADE	Country	Research question	Methods	Population (mean age)	HCP etiology	Measures	Findings
Benchtop perfusion models								
Brydon et al., 1996 [4]	Very low	UK	Does CSF protein or blood affect flow through shunts?	Benchtop perfusion model using variable concentrations of protein and diluted blood suspensions	NA	NA	Valve opening/closing pressure, perfusion pressure, flow	Valve opening and closing pressure were lower with protein solutions compared to control, thus not impairing function. Blood cells adversely affected shunt performance.
Baird et al., 2002 [20]	Very low	USA	Does CSF protein, RBCs, or whole blood affect shunt function?	Benchtop perfusion model using variable concentrations of protein, RBCs, and whole blood dilutions	NA	NA	Valve opening/closing pressure, perfusion pressure	Protein levels have little practical effect on valve function, moderate number of RBC can cause increased variability in valve function, large numbers of RBC uniformly lead to valve failure, prolonged perfusion with dilute whole blood is poorly tolerated, and valve failure is preceded by a period of increased variability in perfusion pressure
Harris et al. 2011 [34]	Very low	USA	Does CSF protein, surface wettability, or flow affect macrophage/astrocyte shunt adhesion?	Benchtop perfusion model using variable protein concentrations and flow rates	n=12 new shunts	NA	Confocal microscopy description of macrophages and astrocytes	Macrophages: oxidation of shunts inhibited binding, while flow and protein were not significant. Astrocytes: increased binding due to flow, while oxidation and protein were not significant.
Cheatle et al., 2015 [22]	Very low	USA	Does CSF protein or blood affect flow through shunts?	Benchtop perfusion model using variable concentrations of protein with new/explanted shunts, variable lengths, with/without valves	NA	NA	Flow resistance	Both new and explanted catheters exhibited a decrease in resistance to flow with higher protein concentrations regardless of the valve used.
Gorelick et al., 2020 [21]	Very low	USA	Does CSF protein increase the risk of shunt failure?	Benchtop perfusion model with upright and supine positions using BSA-augmented human CSF samples	n=6 new shunts	IVH	Flow rate	None of the valves showed evidence of obstruction after 30 days of exposure to CSF containing 5g/L of protein. Variability in flow rates was greatest during supine trials with increased protein.
Qi et al., 2023 [23]	Very low	USA	How to develop an in vitro model reliably recreating proximal ventricular shunt occlusion?	Benchtop perfusion model with chalaza and vitelline membranes as occluding agents to simulate choroid plexus	NA	NA	Blockage	Consistent occlusion was achieved in 5-minute trials using 0.9g of vitelline membrane, which consists of a thin, superficial layer of extraembryonic ectoderm. Future studies may use this model to test occlusion-resistant shunt designs and deobstruction techniques.
Explant analysis								
Gower et al., 1984 [24]	Very low	USA	What cells and debris can be found on functioning vs malfunctioning shunts?	SEM, TEM	n=20 shunts n=12 failure n=8 elective lengthening age=8.3y	Mixed	SEM/TEM description of cells and proteinaceous debris	Functioning shunts: proteinaceous debris forming plaques in areas, few cells, and no platelets. Malfunctioning shunts: a greater number of cells overall, cellular clumps containing platelets, and cells with foot processes attached to the shunt.
Brydon et al., 1998 [25]	Low	UK	What is the quantity and nature of proteins adsorbed to shunts, and could accumulation cause obstruction?	SEM, protein assay	n=102 shunts n=86 permanent n=16 temporary age=children	Mixed	Eluate protein, SEM description of protein, eluate protein bands	Elutable protein was too low to measure despite 20 shunts from patients with CSF protein >1.0g/L. SEM found a thin film of protein on 15% of shunts with no focal protein accumulation. Electrophoresis of eluate found albumin (100%), gamma-globulin (70%), and transferrin (61%) among others. Western blot for IgG, IgM, IgA, fibrinogen, and fibronectin were negative.
Sgouros et al., 2004 [26]	Very low	UK	Do valves of implanted shunts degrade over time due to the deposition of debris?	SEM, EDX microanalysis for chemical composition	n=3 new n=16 explant	NA	SEM description of protein, EDX microanalysis chemical identification	All explanted valves had extensive deposits in all surveyed areas, deposits found as early as two weeks after implantation, and some valves had a film covering the entire outlet. Identity of deposits were sodium and chloride, and occasionally calcium. In all infected and some noninfected valves, carbon was found, indicating protein.
VandeVord et al., 2004 [27]	Low	USA	Could immune response to proteins attached to the shunt, or the shunt itself, contribute to shunt failure?	Protein assay, dot plot for antibodies, anti-polymer antibody assay	n=39 patients n=24 sterile n=8 infection n=7 elective age=10.5y	NA	Eluate protein, autoantibody response	Sterile shunt malfunction explants exhibited a higher rate of protein deposition and these patients had increased levels of autoantibodies to extracted surface proteins. Some individuals develop antibodies to polymeric substances that cross-react with partially polymerized acrylamide.
Czernicki et al., 2009 [5]	Very low	Poland	What can be found inside malfunctioning shunts?	SEM	n=15 shunts	NA	SEM description of shunt surface, protein, and cells	SEM showed shunt inner surfaces were ragged, even among unused shunts, enabling webs of attached collagen fibrils. Within these webs were aggregates of RBC, WBC, platelets, lymphocytes, mast cells, and macrophages. Abnormal RBCs were found.
Hanak et al., 2016 [6]	Very low	USA	What is the cellular mechanism underlying shunt obstruction?	Confocal microscopy with labeled astrocytes, microglia, and choroid plexus	n=36 shunts age=7.2y	Mixed	Confocal microscopy description of astrocytes, microglia, and choroid plexus	Cell attachment was ubiquitous regardless of grossly visible tissue. Among shunts implanted in less than two months, microglia were dominant. Astrocytes were dominant among shunts implanted for longer and served as an interface for secondary attachment of ependymal cells and choroid plexus.
CSF analysis								
Hislop et al., 1988 [35]	Very low	UK	Does elevated CSF protein increase the risk of early shunt failure with PHVD?	CSF analysis of early vs late shunt failure	n=19 patients n=11 early n=8 late age=60d	PHVD	CSF protein	Compared to shunts surviving longer than 6 months, early shunt failure was associated with higher CSF protein and lower weight at surgery.
Pittman et al., 1994 [30]	Very low	USA	Is ETO associated with an immune response leading to shunt malfunction?	CSF analysis, CBC for peripheral eosinophilia, CSF spectrophotometry for ETO/metabolites, western blot for IgE against ETO-treated albumin	n=7 patients age=9.6y	Mixed	CSF: eosinophils, ETO metabolite, culture; peripheral eosinophils, IgE	ETO metabolites were found in CSF months after implantation, 5/7 patients had CSF eosinophilia, 0/7 patients had peripheral eosinophilia, 2/7 patients had detectable serum IgE against albumin-ETO and experienced multiple shunt malfunctions without evidence of infection.
Brydon et al., 1995 [7]	Very low	UK	Does elevated CSF protein affect viscosity enough for shunt failure?	CSF analysis	n=86 patients age=1y, 1m	Mixed	CSF: protein, viscosity	High CSF protein does not greatly affect CSF viscosity. The most viscous CSF likely encountered reduces CSF flow by 6.9% through a shunt compared to the least viscous CSF.
Brydon et al., 1995 [29]	Very low	UK	Does elevated CSF protein affect surface tension and contact angle enough for shunt failure?	CSF analysis	n=27 patients age range=2d-28y	Mixed	CSF: protein, surface tension, contact angle	Both CSF surface tension (affecting valve opening) and contact angle (affecting bacteria attraction to shunt) decreased with an increase in protein concentration until 1g/L, in which protein had little effect.
Bloomfield et al., 1998 [28]	Very low	Australia	Does CSF composition affect viscosity?	CSF analysis	n=23 patients age=43.6y	NA	CSF: protein, viscosity, RBC, WBC, glucose, IgG, albumin	High CSF protein or cell concentration does not significantly affect CSF viscosity. Unlikely that shunt failure is due to increased CSF viscosity.
Mangano et al., 2005 [1]	Very low	USA	Does elevated CSF protein increase the risk of PV/NPV failure?	CSF analysis of PV vs NPV failure	n=189 patients n=35 NPV fail n=48 PV fail mean age=5.9y	Mixed	CSF protein	No significant differences were identified when CSF protein levels and specific malfunction types were compared within the PV and NPV groups.
Rammos et al., 2009 [11]	Very low	USA	Does CSF composition increase the risk of shunt failure with aneurysmal SAH?	CSF analysis	n=80 patients age=60.8y	Aneurysmal SAH	CSF: protein, RBC	Among adult patients with aneurysmal SAH, conversion of EVD to VPS irrespective of CSF protein and RBC counts yielded 3/80 shunt obstructions and 0/80 shunt infections. Using the same EVD site for conversion to VPS can be done safely.
Kang et al., 2010 [12]	Very low	Korea	Does early shunt placement with elevated CSF protein increase the risk of shunt failure with SAH/IVH	CSF analysis of sedimentation vs frank IVH	n=33 patients age=57.8y	Aneurysmal SAH (Fisher grade 3/4)	CSF protein, IVH volume, and ratio	Among patients with aneurysmal SAH, early EVD weaning (4-5 days) and VPS placement yielded a 6.1% revision rate and no shunt infections despite the presence of CSF protein and blood.
Heidemann et al., 2011 [31]	Low	USA	Is eosinophil activation and release of cationic proteins associated with shunt failure?	CSF analysis of eosinophilia vs no eosinophilia	n=56 patients age=113.6m	Mixed	CSF: eosinophils, ECP, EDN	Among patients with sterile shunt malfunction, 17% had CSF eosinophilia, which was not associated with more revisions within 6 months. However, patients with higher ECP did have significantly more revisions within 6 months.
Harris et al., 2021 [32]	Low	USA	Are cytokines and MMPs associated with shunt failure?	CSF multiplex ELISA for cytokines and MMPs	n=38 patients age=8.1y	Mixed	CSF: pro-/anti-inflammatory cytokines (n=10/n=2) and MMPs (n=4)	Among shunts implanted for three months or less resulting in obstruction, there was increased IL-6, IL-8, and MMP-7. Patients with 0-2 past revisions had higher IL-10, IL-6, IL-8, MMP-7, and MMP-9. Differences were also found among etiological and age subgroups.
Khodadadei et al., 2022 [33]	Very low	USA	What are the phenotypes of astrocytes found on shunts?	qPCR/RNA in-situ hybridization for pro-/anti-inflammatory astrocytes (A1/A2), ELISA for cytokines, benchtop model of astrocyte growth modulated by cytokines	n=10 patients	Any	EMP1 and C3 expression, inflammatory cytokines (n=8), A1/A2 activity and adhesion	Shunt obstruction vs survival had a significantly higher proportion of A2 astrocytes (anti-inflammatory). Shunt obstruction also showed higher levels of IL-6, which is pro-A2 and induces the proliferation of astrocytes. The Benchtop model demonstrated that cytokine-neutralizing antibodies prevented the activation of resting astrocytes into A1 and A2 phenotypes.
CSF analysis included in the meta-analysis								
Brydon et al., 1996 [13]	Low	UK	Does elevated CSF protein increase the risk of shunt obstruction?	Prospective, CSF analysis of shunt obstruction vs survival	n=103 shunts n=12 failure n=91 survival age=3.9y	Mixed	CSF: total protein, protein classification	CSF protein did not differ significantly between patients with shunt obstruction vs survival, or shunt infection vs survival.
Ambekar et al., 2011 [8]	Low	India	Does CSF composition increase the risk of shunt failure with TB meningitis?	Case-control, CSF analysis of shunt failure vs survival	n=207 shunts n=70 failure n=137 survival age=15y	TB meningitis	CSF: protein, glucose, cell count	CSF protein was significantly higher among patients with shunt malfunction. CSF protein >200mg/dL was associated with a four times greater risk of shunt malfunction compared to CSF protein <100mg/dL.
Fulkerson et al., 2011 [14]	Low	USA	Does CSF composition increase the risk of shunt failure with PHH?	Case-control, CSF analysis of shunt failure vs survival	n=58 patients n=19 failure n=39 survival mean age=26.5w	PHH	CSF: protein, RBC, WBC, glucose	There were no statistical relationships between shunt failure/infection and CSF protein, RBC, WBC, or glucose. A previous CNS infection prior to shunt insertion was a risk factor for shunt obstruction but not infection.
Bir et al., 2017 [3]	Low	USA	Does elevated CSF protein increase the risk of shunt failure with intracranial meningiomas?	Case-control, CSF analysis of shunt failure vs survival	n=48 patients n=13 failure n=35 survival age=64y	Intracranial meningioma	CSF protein	CSF protein did not differ significantly between shunt failure vs survival. Male patients had longer shunt survival than female patients. Predictors of requiring a shunt include age >65 years, tumor in the posterior fossa, tumor size, and Simpson resection grades II to IV
Kamat et al., 2018 [9]	Low	South Africa	Does CSF composition increase the risk of shunt obstruction with TB meningitis?	Case-control, CSF analysis of shunt obstruction vs non-obstruction	n=590 patients n=170 obstruction n=420 survival age=27.2y	TB Meningitis	CSF: protein, glucose, RBC, lymphocytes, neutrophils	CSF protein was significantly higher among patients with shunt obstruction vs survival. CSF protein did not differ significantly between ventricular vs lumbar samples.
Richetta et al., 2019 [10]	Low	Israel	Does CSF composition increase the risk of shunt failure with brain tumors?	Case-control, CSF analysis of shunt failure vs survival	n=89 shunts n=23 failure n=66 survival age=64y	Brain tumors	CSF: protein, WBC, RBC	CSF protein was significantly higher among patients with brain tumors vs other etiologies. Valve failure vs other malfunction was associated with a three-fold higher CSF protein.
Kaestner et al., 2021 [15]	Low	Germany	Does CSF composition increase the risk of shunt failure?	Case-control, CSF analysis of shunt obstruction vs survival	n=274 patients n=32 failure n=242 survival age=50.4y	Mixed	CSF: protein, WBC	CSF WBC counts but not protein was associated with valve occlusion overall. 13.6% of patients had CSF protein between 100mg/dL and 1000mg/dL and did not develop shunt malfunction. Persistently high CSF protein at the time of shunt revision was associated with early valve obstruction.
Dong et al., 2022 [16]	Low	China	Does CSF composition increase the risk of shunt failure with HIV-negative cryptococcal meningitis?	Case-control, CSF analysis of shunt failure vs survival	n=76 patients n=12 failure n=64 survival age=43.9y	Cryptococcal meningitis (HIV negative)	CSF: protein, WBC, blood glucose ratio, chloride ion, Cryptococcal count	CSF protein did not differ significantly between shunt failure vs survival. Ventricular CSF blood glucose ratio was lower in shunt failure. All CSF parameters differed significantly between lumbar and ventricular samples, including CSF protein. Ventriculomegaly was associated with higher ventricular CSF protein.

Among the eight studies included in the meta-analysis, seven were case-control [3,8-10,14-16] studies and one was a prospective study [13]. Two studies included neonates and early pediatric patients [13,14]. Across the studies, 48.2% of the participants were female. The minimum follow-up period was two months (mean=18.4 months). Studies have used different definitions of shunt failure; however, these differences have been mitigated; shunt obstruction is the most common type of failure. Four studies defined failure as any type [3,10,14,16]; three studies defined failure as obstruction only [9,13,15]; one study defined failure as excluding infection and malposition [8]. The failure rates among the included studies ranged from 11.65% to 15-32.76% [14], with a mean of 21.15%. Studies investigating populations with a specific HCP etiology have included TB meningitis [8,9], HIV-negative cryptococcal meningitis [16], post-hemorrhagic [14] meningioma [3], and brain tumors [10]. Two studies included patients with multiple etiologies [13,15]. At the univariate level, only two studies showed significantly higher CSF protein levels in patients with shunt failure than in those with survival [8,9].

Among the 31 studies included in the systematic review, six were benchtop perfusion models that studied perfusion pressure, valve opening/closing pressure [4,20], flow rate [21], resistance to flow [22], and percentage blockage [23]. Six studies involved explant analysis using scanning electron microscopy (SEM) [5,24-26], transmission electron microscopy [24], confocal microscopy [6], energy-dispersive X-ray spectroscopy [26], and protein assays [25,27]. Nineteen studies were CSF analyses and measured a wide variety of CSF parameters in addition to CSF proteins, such as viscosity [7,28], surface tension, contact angle [29], ethylene oxide metabolites [30], protein identification [13], eosinophil cationic protein and eosinophil-derived neurotoxin [31], cytokines [32,33], and matrix metalloproteinases (MMPs) [32].

Risk of Bias

**Figure 2:**
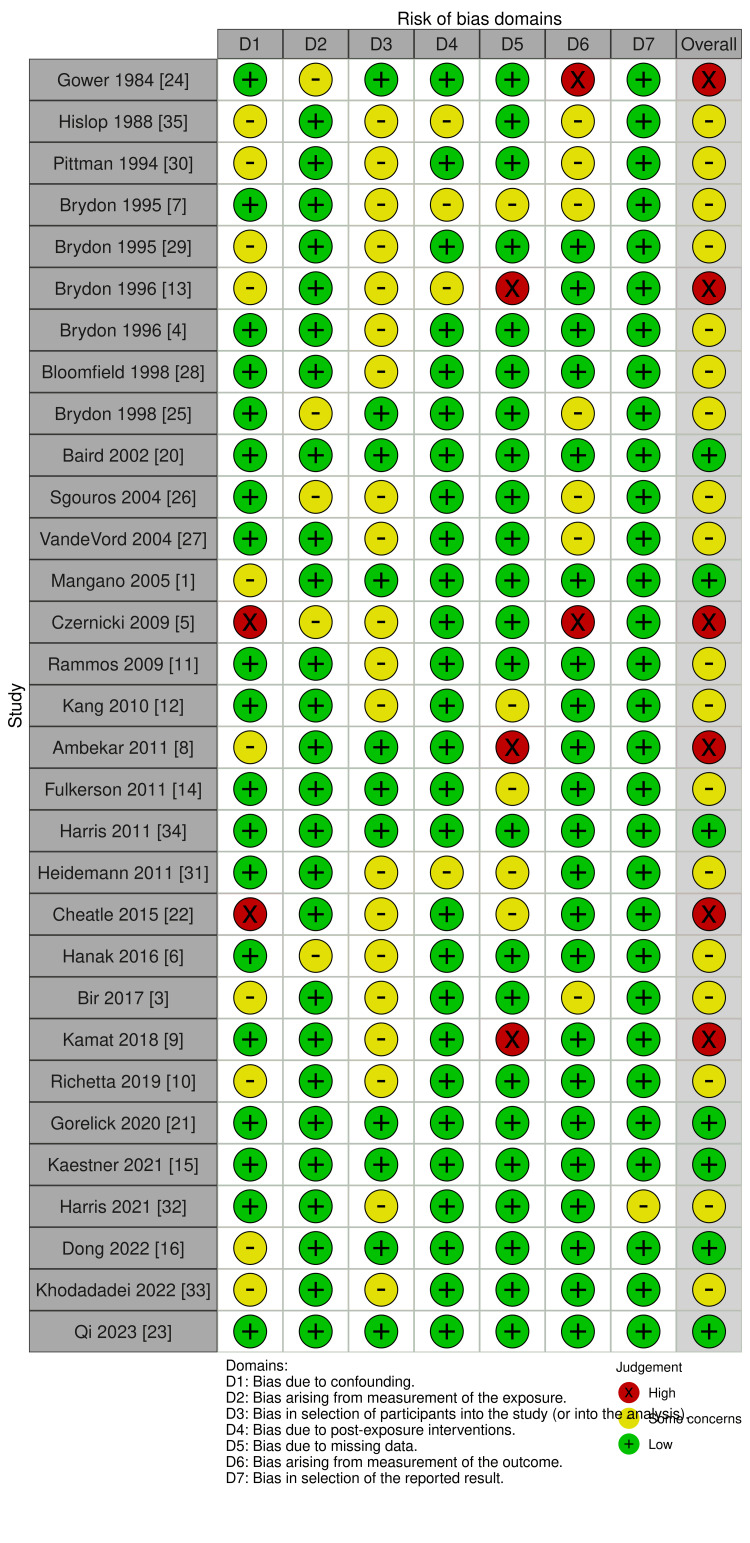
Risk of bias traffic light plot The risk of bias was assessed using ROBINS-E. ROBINS-E: Risk Of Bias In Non-randomized Studies - of Exposures Reference: Brydon 1995a [7]; Brydon 1995b [29]; Brydon 1996a [13]; Brydon 1996b [4]

**Figure 3:**
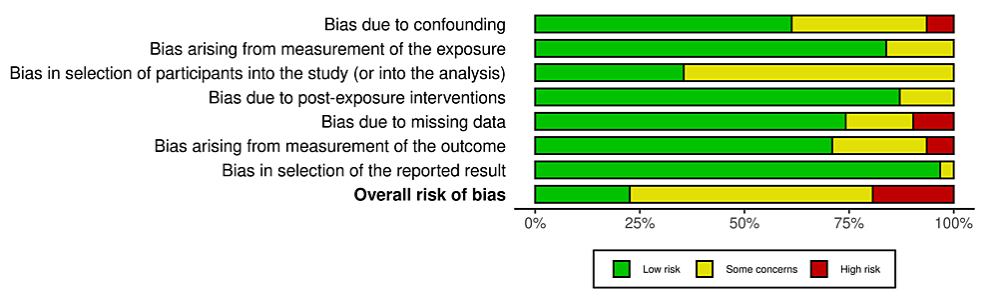
Risk of bias summary plot The risk of bias was assessed using ROBINS-E. ROBINS-E: Risk Of Bias In Non-randomized Studies - of Exposures

The risk of bias was assessed using the ROBINS-E (Figures 2, 3). This analysis yielded 7 studies that were low risk, 18 studies with some concerns, and 6 studies with high risk. Two studies were considered to have a high risk of confounding owing to the use of explanted shunts without providing sufficient information about patient history [5,22]. Three studies were considered high-risk owing to missing SD values [8,9,13]. Two studies were considered high risk because the outcome measures were nonblinded qualitative SEM analyses [5,24]. Overall, most concerns arose from (1) confounding due to a lack of description and univariate analyses, and (2) participant selection due to a lack of description.

Meta-Analysis Results

**Figure 4:**
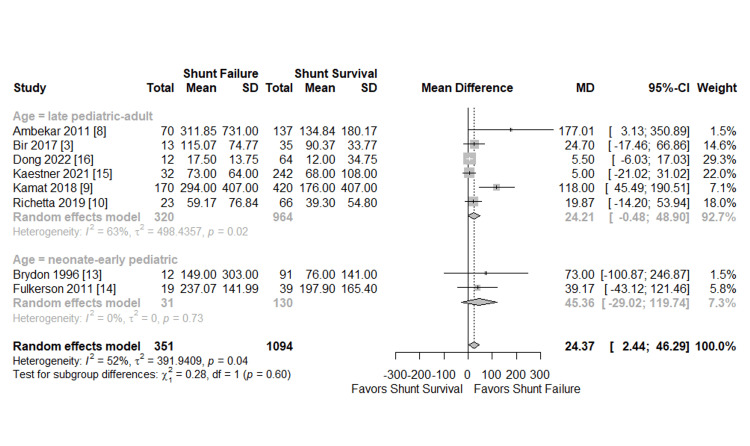
Meta-analysis etiology subgroup Forest plot subgroup analysis by hydrocephalus (HCP) etiology of perioperative cerebrospinal fluid (CSF) protein level among shunt failure vs survival groups. Studies were subgrouped by HCP etiology because while the authors aim to study the effect of CSF protein on shunt failure, the true cause is unlikely to be univariate and CSF composition can vary widely depending on HCP etiology. SD: Standard deviation; MD: Mean difference; CI: Confidence interval

**Figure 5:**
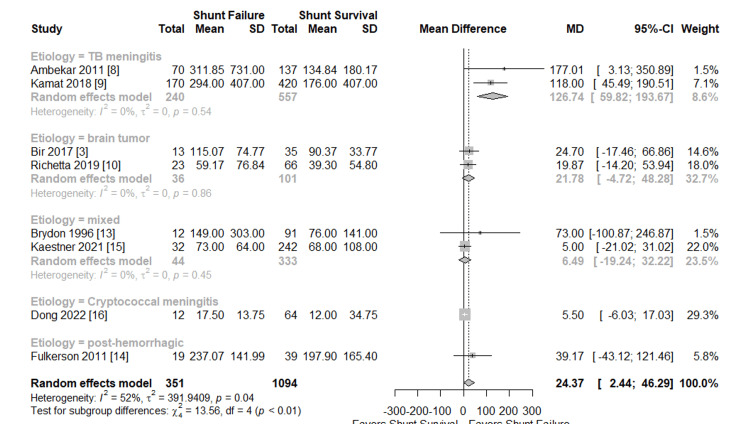
Meta-analysis of age subgroup Forest plot subgroup analysis by the age of perioperative cerebrospinal fluid (CSF) protein level among shunt failure vs survival groups. Studies with neonates to early-pediatrics were subgrouped because this population faces different etiologies and complications than adults with ventriculoperitoneal shunt (VPS). The remaining studies were categorized as late pediatric adults. SD: Standard deviation; MD: Mean difference; CI: Confidence interval

Meta-analyses of perioperative CSF protein levels and shunt failure are presented in Figures 4, 5, respectively. Eight studies with 1,445 patients (351 shunt failures and 1094 shunt survivals) were included in the analysis [3,8-10,13-16]. The effect measure was the MD, with a positive value indicating shunt failure. Overall, the MD in CSF protein was 24.37 mg/dL favoring shunt failure, which was significant with the 95% CI not overlapping zero (2.44-46.29 mg/dL). This model represented substantial heterogeneity in magnitude (I2=52%) and was statistically significant (p=0.04). This was anticipated prospectively; subgroup analysis was planned for age and etiology.

Meta-Analysis Subgroup: Age

A subgroup analysis was performed based on age (Figure 4). Two studies with neonates to early pediatrics were subgrouped and the CSF protein MD=45.36 mg/dL was not significant (95% CI, -29.02-119.74) [13,14]. However, this subgrouping addressed the issue of heterogeneity with I2=0%. The remaining six studies were subgrouped as late pediatric to adult and were also found to be not significant, with MD=24.21 mg/dL (95% CI, -0.48, 48.90) [3,8-10,15,16]. Additionally, the latter subgroup may represent significant and substantial heterogeneity, with I2=63% and p=0.02. It was hypothesized that this heterogeneity stemmed from etiological differences among these patients, which was further explored in the subsequent subgroup analysis by etiology. The chi-squared test for subgroup differences did not find significant differences between the neonate and early pediatric and late pediatric-adult studies (p=0.60).

Meta-Analysis Subgroup: HCP Etiology

A subgroup analysis was also performed to determine the etiology of HCP (Figure 5). Two studies of patients with TB meningitis were found to be significant with MD=126.74 mg/dL (95% CI, 59.82-193.67), and heterogeneity was minimal (I2=0%) [8,9]. Two studies of patients with brain tumors were found to be not significant with MD=21.78 mg/dL (95% CI, -4.72-48.28) and heterogeneity was minimal (I2=0%) [3,10]. Two studies of patients with an assortment of etiologies (labeled “mixed”) were found to be not significant with MD=6.49 mg/dL (95%CI, -19.24-32.22) and heterogeneity was minimal (I2=0%) [13,15]. Finally, one study with HIV-negative cryptococcal meningitis and post-hemorrhagic HCP were given independent subgroups and neither study showed a significant MD (cryptococcal meningitis MD=5.50 mg/dL, 95% CI=-6.03-17.03; posthemorrhagic MD=39.17 mg/dL, 95% CI=-43.12-121.46) [14,16]. The chi-square test revealed significant differences between the etiological subgroups (p<0.01).

Meta-Analysis: Risk of Bias

Using ROBINS-E, we found that five studies had some concerns of bias due to confounding factors, often resulting from the retrospective design and univariate analyses that did not adequately control for confounders (i.e., age, etiology, shunt/valve type, failure type, and follow-up period) [3,8,10,13,16]. Four studies had concerns of bias due to participant selection, often resulting from a lack of description of how patients were chosen for selection [3,9,10,13]. Three studies had a high risk of bias due to missing data resulting from missing SD [8,9,13].

Sensitivity Analysis

Sensitivity analysis was performed to evaluate the robustness of these meta-analyses despite the required estimation of SD in three of the studies [8,9,13]. To test this, the meta-analysis was performed for an additional time excluding these three studies. The association between CSF protein and shunt failure was not significant with MD=8.09 mg/dL (95% CI=-1.63-17.82). Notably, the heterogeneity for this model was minimal despite combining multiple age and etiological groups, with I2=0%.

Publication Bias

The risk of publication bias was assessed using a funnel plot and metabias R function. Asymmetry was noted in studies with a larger standard error and MD. The Egger regression test yielded p=0.01, indicating publication bias. However, owing to missing data, large estimated SD values were included in the analysis. Therefore, a second funnel plot was created, excluding studies with missing SD data, and the Egger regression test yielded p=0.04, thus indicating publication bias: a lack of smaller studies reporting low MD of CSF protein in the shunt failure vs. survival groups.

Certainty

The certainty of evidence was assessed using the GRADE. None of the studies included in the meta-analysis were randomized controlled trials, the studies began the analysis with low-grade evidence. Each outcome was assessed across the domains of limitations, inconsistency, indirectness, imprecision, and publication bias with no grade decrease. This low grade of evidence indicates that further research is likely to have an important impact on our confidence in the estimate of the effect, and is likely to change the estimate [19].

Discussion

Synthesis of Meta-Analysis

A meta-analysis of eight studies found that a higher perioperative CSF protein level significantly favored shunt failure. However, the sensitivity analysis demonstrated that this finding lacked robustness when excluding three studies [8,9,13] with missing SD data; the meta-analysis of the five remaining studies did not show significance. The overall model showed substantial and significant heterogeneity, and subgroup analyses according to age and HCP etiology yielded minimal heterogeneity. However, these subgroups generally include only two studies per category. Only the TB meningitis subgroup showed significant MD with higher CSF protein levels, thereby favoring shunt failure.

Given the lack of robustness due to missing data, high degree of heterogeneity, scarcity of studies, and low GRADE of evidence, the true relationship between CSF protein levels and shunt failure remains unknown. The remainder of this discussion aims to synthesize the findings from the systematic review and suggest future directions for research.

Synthesis of Benchtop Perfusion Models

The assertion that elevated CSF protein levels increase the risk of shunt failure has long been assumed, but lacks robust evidence [4]. Two main hypotheses have been proposed to explain the potential role of CSF proteins in shunt failure: (1) an intrinsic property of proteins directly inhibiting shunt flow and (2) an intrinsic property of proteins that enables secondary attachment leading to shunt obstruction.

One hypothesis suggested that proteins increase the viscosity of the CSF, thereby impeding flow. However, studies have shown that high protein concentrations have minimal impact on CSF viscosity [28], with only a 7% difference in flow between protein concentrations above 500 mg/dL and below 45 mg/dL [7,21]. Additionally, according to Poiseuille’s equation, viscosity affects laminar flow to the same degree as the catheter length, which is rarely considered [7].

Another hypothesis proposed that proteins increase the surface tension, hindering the opening of the shunt valve [29]. However, the relationship among CSF proteins, surface tension, and opening/closing pressures is complex and contradictory. Some studies have suggested that higher CSF protein levels are associated with lower surface tension [22,29], while others have suggested the opposite depending on the CSF protein level [20].

A third hypothesis suggested that proteins facilitate the secondary attachment of cells (i.e., macrophages and astrocytes) leading to shunt obstruction. Increasing CSF protein levels result in greatly increased macrophage adherence under static conditions; astrocytes gain increased adherence under fluidic conditions [34]. These findings indicate a role for proteins in secondary cell attachment, which may depend on cell-specific properties.

Synthesis of Explant Analyses

Explant analyses use various imaging techniques to examine the debris lining the inner surfaces of explanted shunts. Early analyses showed that both functional and failed shunts developed a proteinaceous layer of debris, with failed shunts exhibiting greater cellular involvement [24]. SEM electron microscopy revealed reactive cells with foot processes attached to the inner surfaces of the shunt. It has been suggested that the breakage of an internal protein layer may facilitate cellular migration on the silicone surface of the shunt [24]. Additionally, SEM analysis demonstrated that the inner surfaces of the shunt catheters, both new and explanted, lacked smoothness, which promoted collagen fibril attachment and subsequent entrapment of cells [5]. Further studies have visualized the attachment of astrocytes, serving as an interface for the attachment of ependymal cells and choroid plexus, contributing to shunt obstruction [6]. Therefore, it is hypothesized that proteins may attach to imperfections in the shunt surface with secondary attachment of cells, particularly astrocytes, and tertiary attachment of ependymal cells and choroid plexus responsible for obstruction.

The temporal progression of debris accumulation was investigated, thus revealing the development of mineral deposits (i.e., Ca, Mg, Na, K, and Cl) ranging from small crystals to large cobblestones [26]. Similarly, changes in cellular adherence were observed with early migration of microglia, followed predominantly by astrocyte adherence over time [6]. The early involvement of microglia in shunts is notable in the context of Gower's early hypothesis, which links cellular migration and shunt adherence to a delayed immune hypersensitivity response observed with other silicone implants [24]. Further investigation revealed increased levels of autoantibodies against autologous silicone-bound surface proteins in cases of sterile shunt failure, suggesting a potential role for the immune response in shunt obstruction [27].

Synthesis of CSF Analyses

Despite the conventional wisdom, the association between CSF protein levels and shunt failure remains controversial. Some studies have found no significant difference in CSF protein levels between shunt failure and survival groups and argue against delaying VPS placement [11,12]. Other studies reported an association between elevated CSF protein levels and shunt failure [8-10]. One study concluded that CSF protein levels > 200 mg/dL significantly increased the risk of shunt failure [8]. It was also found that elevated CSF protein at shunt placement was significantly associated with early shunt failure [35], especially if persistently elevated [15].

In addition to protein levels, studies have investigated the identity of specific proteins in CSF and their association with shunt failure [13,31-33]. The proteins detected in explanted shunts primarily include albumin, gamma globulin, transferrin, and, to a lesser extent, haptoglobin, tau protein, alpha-1 antitrypsin, and prealbumin [13,25]. Furthermore, a study investigating the role of cytokines and MMPs found that early shunt failure was associated with elevated IL-6, IL-8, and MMP-7 [32]. Future work also found elevated IL-6 levels are associated with shunt obstruction, which activates the anti-inflammatory phenotype of astrocytes (A2) [33]. Since obstructed shunts have significantly more A2, cytokine-neutralizing antibodies may offer therapeutic benefits [33].

Limitations

This meta-analysis and systematic review have several limitations. First, most studies included in the analysis relied on univariate analysis of CSF protein levels without considering important confounders. The lack of multivariate analysis limits the interpretation of the findings. Second, a potential publication bias was identified, with studies reporting lower MDs in CSF protein levels between the shunt failure and survival groups being less likely to be published. Third, the analysis suffers from missing data, which affects the robustness of the results. Additionally, the overall certainty of evidence (GRADE) was low due to the predominance of case-control study designs, and there was substantial and significant heterogeneity. Finally, the lack of standardized language and definitions in this field further complicates the interpretation and comparison of results.

Implications for future research

Given the limitations of the current meta-analysis, further research is needed to better understand the relationship between perioperative CSF protein levels and shunt failure. Standardizing the language and definitions in this field would enhance communication and outcome comparability. Multivariate approaches should be employed to minimize confounding factors, particularly when considering important factors such as age and etiology. Investigating CSF protein thresholds and the associated odds ratios of shunt failure would yield more clinically relevant conclusions. Furthermore, exploring the specific mechanisms underlying protein attachment and shunt obstruction is crucial for the development of preventive strategies. Future research should explore the hypothesis developed from the synthesis of current evidence that proteins may attach to imperfections on the shunt surface, leading to secondary attachment of cells, particularly astrocytes, and tertiary attachment of ependymal cells and the choroid plexus, which ultimately causes obstruction. Finally, investigating the possible immune response underlying the pathogenesis of shunt obstruction and evaluating the potential therapeutic role of cytokine-neutralizing antibodies warrant further investigation.

## Conclusions

This meta-analysis found that higher perioperative CSF protein levels significantly favored shunt failure; however, sensitivity analysis demonstrated that this finding lacked robustness when excluding studies with missing data. Given the lack of robustness, high degree of heterogeneity, scarcity of studies, and low certainty of evidence (GRADE), the true relationship between CSF proteins and shunt failure remains unknown. Additional information, such as the duration of ventriculostomy before VPS placement, may offer nuances to this topic; at least one may assume that a functioning ventriculostomy in the face of high protein levels may imply that the patient will tolerate a VPS with similar protein levels. Future studies should use multivariate analysis to control confounding factors and investigate CSF protein thresholds to guide clinical decision-making. Future research should investigate the specific pathogenesis underlying protein attachment and shunt obstruction. A hypothesis developed from a synthesis of existing research is presented. Proactive studies of ventriculostomy obstruction in patients with high CSF protein levels may also be helpful.
